# Hepatitis C Knowledge and Self-Reported Testing Behavior in the General Population in China: Online Cross-Sectional Survey

**DOI:** 10.2196/39472

**Published:** 2023-12-11

**Authors:** Yin Liu, Juan Su, Xiaoyang Wang, Huifang Xu, Hong Wang, Ruihua Kang, Liyang Zheng, Yixian Wang, Chunya Liu, Yiping Jing, Shaokai Zhang

**Affiliations:** 1 Department of Cancer Epidemiology The Affiliated Cancer Hospital of Zhengzhou University (Henan Cancer Hospital) Zhengzhou China; 2 Yinchuan Hospital of Stomatology Yinchuan China

**Keywords:** HCV, hepatitis C virus, knowledge, testing behaviors, general population, cross-sectional, online survey, testing, screening, patient education, China, Chinese, patient education, health education, hepatitis, viral disease, viral infection, communicable disease, liver

## Abstract

**Background:**

The World Health Organization has proposed a worldwide target of eliminating hepatitis C virus (HCV) by 2030. A better understanding of HCV, testing behaviors, and associated factors in the general population is essential.

**Objective:**

This study aimed to assess HCV knowledge, self-reported HCV testing behavior, and willingness to undergo HCV screening in the general Chinese population.

**Methods:**

A cross-sectional online survey of the general Chinese population aged ≥15 years was conducted from November 2021 to May 2023. Participant characteristics were assessed based on their knowledge level and uptake of HCV testing. Participants ever having heard of HCV were recognized as being aware of HCV and asked additional HCV knowledge questions using a brief, validated 9-item scale. Participants with 0-3 points and who were unaware of HCV were categorized as having poor knowledge, and those with 4-6 points and ≥7 points were categorized as having fair and good knowledge, respectively. Participant uptake of HCV testing, testing results, reasons for undergoing or not undergoing HCV testing, and willingness to undergo HCV screening were collected through self-reports. Ordinal and binary logistic regression analyses were used to assess factors associated with the HCV knowledge level and the uptake of HCV testing, respectively.

**Results:**

A total of 1491 valid participants’ questionnaires were included. Of these, 714 (47.6%) participants were aware of HCV. The proportion of participants with poor, fair, and good HCV knowledge was 63.4% (945/1491), 9.3% (139/1491), and 27.3% (407/1491), respectively. A total of 465 (31.2%) participants reported ever undergoing HCV testing, and 4 (0.9%) were anti-HCV antibody positive. Most participants were tested for HCV following blood donation (353/465, 75.9%). The most common reasons for not undergoing HCV screening were a lack of HCV awareness (665/1026, 64.8%), followed by a low self-perceived risk of infection (176/1026, 17.2%). Of 1026 participants who had never undergone HCV testing, 937 (91.3%) were willing to undergo HCV screening if universal screening was provided at no cost. The HCV knowledge level was positively associated with the HCV testing rate. Participants who were less educated, lived in rural areas, resided in West China, and were currently alcohol drinkers had lower HCV knowledge and reduced odds of having undergone HCV testing. In contrast, participants with a blood donation history and a family history of hepatitis B virus or HCV infection had higher HCV knowledge and increased odds of prior testing. Participants aged ≥60 years had lower knowledge, and women had reduced odds of having undergone previous HCV testing.

**Conclusions:**

The general population of China has low HCV knowledge and testing rate. There is an urgent need for enhanced HCV awareness and scaled-up HCV screening and treatment. Individuals who are less well educated, reside in less-developed areas, currently drink alcohol, and are female should be prioritized for health education and interventions.

## Introduction

Hepatitis C virus (HCV) infection is a major cause of acute and chronic liver diseases (eg, liver cirrhosis and hepatocellular carcinoma) worldwide and is linked to an estimated 29,000 deaths each year [1]. About 58 million people have HCV, with approximately 1.5 million new infections occurring annually [1]. China has the largest number of patients with HCV infection, accounting for approximately 14% of worldwide HCV infections [2,3].

In 2016, the World Health Organization (WHO) responded to the high worldwide burden of HCV by aiming to reduce new infections by 80% and deaths by 65% by 2030 [4]. Given the lack of an effective vaccine, WHO prioritized improving HCV knowledge and scaling up screening, care, and treatment to meet its targets. Adequate HCV knowledge is critical for the prevention of HCV infection and transmission and for individuals to seek appropriate testing, care, and treatment [5]. In China, studies have explored HCV knowledge among groups at high risk of HCV infection and transmission, including people receiving methadone maintenance treatment (MMT) [6] and men who have sex with men (MSM) [7]. However, no studies have assessed HCV knowledge in the general population. Although most new HCV infections are among high-risk groups, many people are either not aware of or refuse to report risk behaviors because of potential discrimination and therefore remain hidden in the general population. This puts additional people at risk of HCV exposure through contaminated blood, unsafe sexual contact, and mother-to-child transmission [8,9]. A meta-analysis found that the national prevalence of HCV infection in the general Chinese population was about 0.91% [10], constituting a large subgroup with the ability to further spread HCV. Thus, an assessment of HCV knowledge and associated factors in the general Chinese population is important to inform HCV elimination plans.

Scaling up screening, care, and treatment is also critical to achieve WHO targets. People with HCV infection can be easily screened with an anti-HCV antibody test, and more than 95% of these individuals can be cured using highly effective oral direct acting antiviral (DAA) treatment regimens [11]. In China, DAAs have been approved for use against HCV infection since 2017 and have been listed in the medical insurance directory since 2020. However, because HCV is often asymptomatic, only 18% of people with HCV infection are diagnosed, leading to a low treatment rate [12]. As a result, a low HCV testing rate is the main barrier to HCV elimination. In China, HCV screening is primarily limited to high-risk groups [13]. Economic analyses have revealed that screening alongside DAA treatment is cost-effective for both the high-risk population and the general population [14-17]. Thus, this strategy may be necessary to achieve the WHO target. However, there is a lack of information about HCV testing behavior and willingness to undergo HCV screening in the general Chinese population.

This study was conducted to assess HCV knowledge, self-reported HCV testing behavior, and willingness to undergo HCV screening in the general Chinese population to inform policy decision-making aimed at eliminating HCV. The characteristics of patients who have less knowledge and are less likely to undergo testing were also explored.

## Methods

### Design and Setting

A cross-sectional, online survey was conducted in China from November 2021 to May 2023. The convenience sampling method was used to recruit participants. Individuals who lived in China and were ≥15 years of age were eligible for the study. Those who were health care workers, including physicians, nurses, nurse assistants, pharmacists and pharmacy technicians, radiographers, community health workers, health officers, hospital orderlies, and other health care professionals, or health care students, were excluded.

### Participant Characteristics

The following information was collected: (1) sociodemographic characteristics, including age, gender, marital status (married, single/divorced/widowed), years of education (<7 years, 7-12 years, >12 years), ethnicity (Han, minority), occupation (student, not working/unemployed, others), residence (rural, urban), and geographic region (east, central, west) [17]; (2) behavioral characteristics, including alcohol consumption (never, current, ever) and history of blood donation (yes, no); and (3) family history of hepatitis B virus (HBV) or HCV (yes, no) infection.

### HCV Knowledge

Initially, awareness of HCV was determined based on participant responses to the following question: “Have you ever heard of hepatitis C disease or hepatitis C virus (HCV)?” Individuals who replied yes were considered as being aware of HCV and asked additional questions to assess the depth of their HCV knowledge based on surveys conducted previously [18,19]. After meeting the standards for reliability and validity, a brief 9-item scale including questions about the nature of hepatitis C disease and modes of transmission was developed for this study. Correct answers scored 1, and incorrect or “don’t know” answers scored 0. Overall HCV knowledge scores ranged from 0 to 9. Participants were categorized into 3 groups based on their knowledge scores. Those scoring 0-3 points were categorized as having poor knowledge, those scoring 4-6 points were categorized as having fair knowledge, and those scoring 7-9 points were categorized as having good knowledge of HCV. Participants who had never heard of hepatitis C disease or HCV were recognized as being unaware of HCV and categorized as having poor knowledge.

### Participant HCV Testing Behavior and Willingness to Undergo HCV Screening

Participant uptake of HCV testing, testing results, reasons for undergoing or not undergoing HCV testing, and willingness to undergo HCV screening were collected through self-reports.

The uptake of HCV testing was determined from responses to the following question: “Have you ever been tested for HCV? (yes/no)”. Participants who reported yes were asked the following questions: “How many tests for HCV have you undergone?,” “When was the last testing?,” “What was the result of the last testing? (positive/negative/unaware),” and “What was the reason for the last testing?” Participants who reported no were further asked about their main reason for not undergoing HCV testing and whether they were willing to undergo HCV testing if universal screening was provided free of charge. Those not willing to undergo screening were asked to provide the main reason why not.

In China, potential blood donors are required to undergo HCV testing since 1993 [20]. All participants in this study with a history of blood donation after 1993 were informed in advance about undergoing HCV testing in order to reduce any self-reporting biases.

### Data Collection

**Figure 1:**
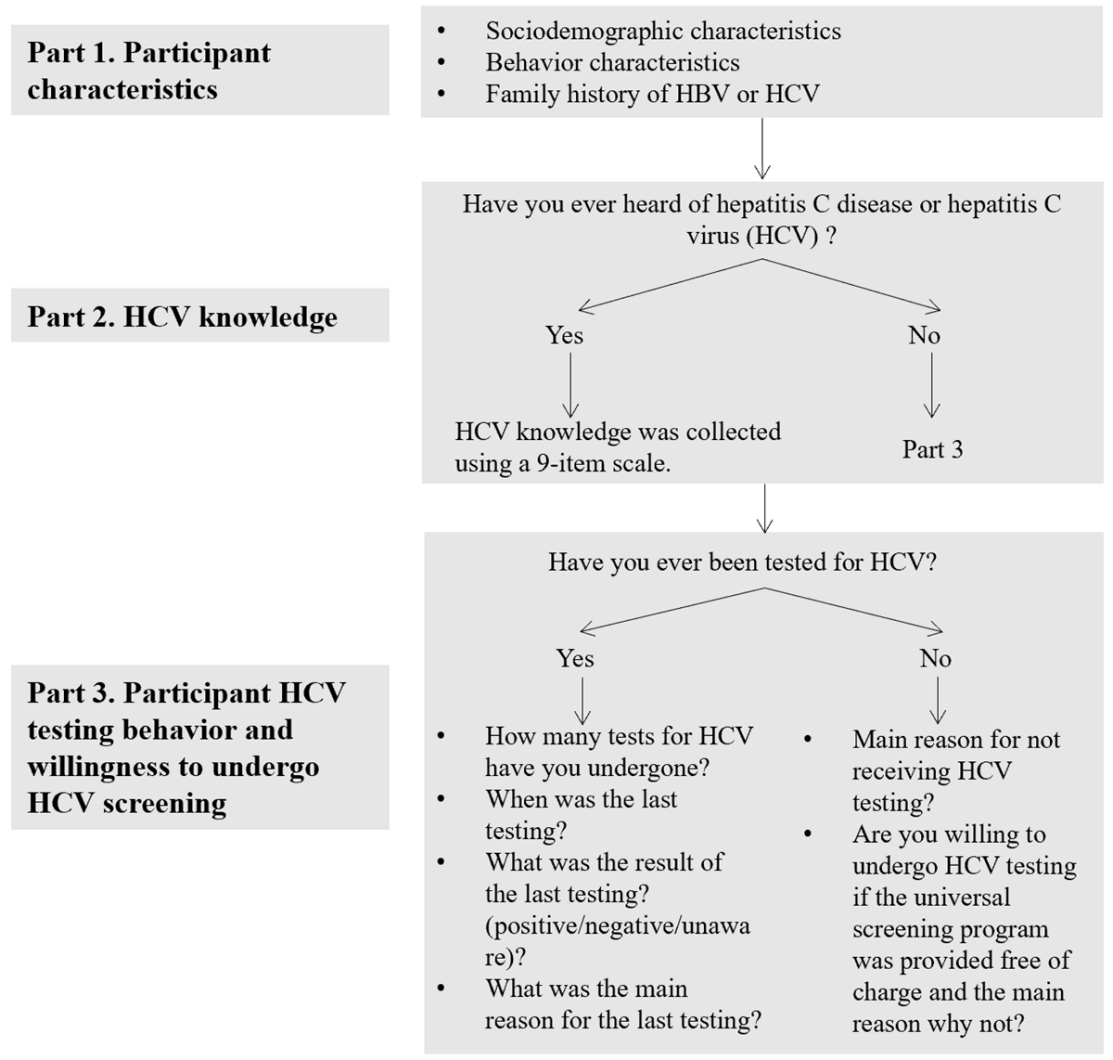
The structure of the questionnaire. HCV: hepatitis C virus.

WeChat and Weibo were the primary method of recruiting eligible participants. An anonymous, self-administered questionnaire was developed that comprised 3 parts. The structure of the questionnaire is shown in Figure 1. Wenjuanxing, the most popular social media platform in China, was used to disseminate the electronic questionnaire.

### Sample Size

The level of HCV knowledge (poor, fair, good) was used as the main outcome to evaluate the sample size, and the following formula was used to determine the multinomial proportions [21]:



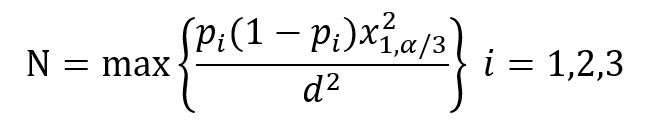



It was assumed that the proportion of participants with poor knowledge (p_1_), fair knowledge (p_2_), and good knowledge (p_3_) was 60%, 15%, and 25%, respectively; the confidence level (1 – α) was 95%, and precision (d) was 5%. Thus, the target sample size was calculated as 551 respondents. Considering a nonresponse rate of 10%, it was determined that at least 613 participants should be enrolled in the study.

### Statistical Analysis

The data collected in Wenjuanxing were exported into Microsoft Excel and then imported into SAS V9.4 for data analysis. The reliability and validity of the HCV knowledge scale were determined. The Cronbach coefficient was calculated to determine reliability. The average variance extracted (AVE) and composite reliability (CR) were calculated for convergent validity. The square roots of the AVE and the absolute values of correlation coefficients between factors were measured for discriminant validity. Confirmatory factor analysis (CFA) was performed for the goodness of fit.

Mean (SD) values were used for normally distributed continuous variables, and medians, the first quartile (Q1), and the third quartile (Q3) were used for nonnormally distributed continuous variables. The frequency distribution and percentage were used to describe categorical variables.

Factors independently associated with HCV knowledge were assessed using univariate and multivariate ordinal logistic regression analyses, with the HCV knowledge level (poor, fair, good) as the dependent variable and participant characteristics as potential independent variables. The proportional odds assumption was assessed, with *P*>.05 being considered statistically significant. Factors independently associated with the uptake of HCV testing were assessed using univariate and multivariate binary logistic regression analyses, with the uptake of HCV testing (yes, no) as the dependent variable and participant characteristics as potential independent variables. A correlation matrix was used to exclude the confounding variables with strong collinearity. Variables with *P*<.1 in univariate models were used in multivariate regression models. Statistical significance was set as *P*<.05. A crude odds ratio (cOR) with 95% CI was reported for univariate models, and an adjusted odds ratio (aOR) with 95% CI wAs reported for multivariate models. Sensitivity analyses were conducted by limiting the data set to participants without a history of blood donation to control for the impact of blood donation on HCV testing behavior.

### Ethical Considerations

Ethical approval was obtained from Henan Cancer Hospital (2023-KY-0139). The electronic questionnaire was written in standard Chinese. The participants received a cover letter informing them of the study aims and procedures and that their participation was anonymous and voluntary. It was assumed that individuals who completed the survey had read the cover letter and consented to participate. To ensure the effectiveness of data collection, participants were required to answer a question to receive the next question. All completed questionnaires were kept private and were accessible only to the study staff responsible for data analysis.

## Results

### Participant Characteristics

A total of 1929 complete questionnaires were obtained, and after excluding those from health care workers or health care students, 1491 (77.3%) participants from 26 provinces were included in the final analysis. The geographical distribution of the included participants is shown in Figure S1 in Multimedia Appendix 1. Characteristics of the included participants are shown in Table 1. Of the 1491 participants, 476 (31.9%), 364 (24.4%), and 651 (43.7%) were from East, West, and Central China, respectively. In total, the average age of the participants was 41.4 (SD 15.4) years. Slightly over half of the participants were female (n=819, 54.9%) and located in urban areas (n=843, 56.5%). Most participants were married (n=1060, 71.1%), educated for >7 years (n=1160, 77.8%), of Han nationality (n=1405, 94.2%), and employed/self-employed (n=1286, 86.3%). About 26.4% (n=394) of the participants currently drank alcohol, 25.3% (n=377) had ever donated blood, and 12.4% (n=185) had a family history of HBV or HCV.

**Table 1:** Characteristics of the included participants (N=1491).

Characteristics		Total	East China		West China		Central China
**Age (years)**							
	<60, n (%)	1230 (82.5)	433 (91.0)	310 (85.2)		487 (74.8)	
	≥60, n (%)	261 (17.5)	43 (9.0)	54 (14.8)		164 (25.2)	
	Mean (SD)	41.4 (15.4)	38.1 (13.9)	38.8 (15.8)		45.2 (15.4)	
**Gender, n (%)**							
	Male	672 (45.1)	204 (42.9)	178 (48.9)		290 (44.5)	
	Female	819 (54.9)	272 (57.1)	186 (51.1)		361 (55.5)	
**Marital status, n (%)**							
	Single/divorced/widowed	431 (28.9)	168 (35.3)	140 (38.5)		123 (18.9)	
	Married	1060 (71.1)	308 (64.7)	224 (61.5)		528 (81.1)	
**Years of education, n (%)**							
	<7	331 (22.2)	81 (17.0)	22 (6.0)		228 (35.0)	
	7-12	440 (29.5)	103 (21.6)	150 (41.2)		187 (28.7)	
	>12	720 (48.3)	292 (61.3)	192 (52.7)		236 (36.3)	
**Ethnicity, n (%)**							
	Han	1405 (94.2)	448 (94.1)	316 (86.8)		641 (98.5)	
	Minority	86 (5.8)	28 (5.9)	48 (13.2)		10 (1.5)	
**Occupation, n (%)**							
	Students	139 (9.3)	32 (6.7)	72 (19.8)		35 (5.4)	
	Employed/self-employed	1286 (86.3)	420 (88.2)	280 (76.9)		586 (90.0)	
	Not working/unemployed	66 (4.4)	24(5.0)	12 (3.3)		30 (4.6)	
**Residence, n (%)**							
	Urban	843 (56.5)	340 (71.4)	264 (72.5)		239 (36.7)	
	Rural	648 (43.5)	136 (28.6)	100 (27.5)		412 (63.3)	
**Alcohol consumption, n (%)**							
	Never	961 (64.5)	318 (66.8)	210 (57.7)		433 (66.5)	
	Current	394 (26.4)	122 (25.6)	106 (29.1)		166 (25.5)	
	Ever	136 (9.1)	36 (7.6)	48 (13.2)		52 (8.0)	
**History of blood donation, n (%)**							
	Yes	377 (25.3)	147 (30.9)	70 (19.2)		160 (24.6)	
	No	1114 (74.7)	329 (69.1)	294 (80.8)		491 (75.4)	
**Family history of HBV^a^ or HCV^b^ infection, n (%)**							
	No	1306 (87.6)	425 (89.3)	322 (88.5)		559 (85.9)	
	Yes	185 (12.4)	51 (10.7)	42 (11.5)		92 (14.1)	

^a^HBV: hepatitis B virus.

^b^HCV: hepatitis C virus.

### Reliability and Validity of the HCV Knowledge Scale

The HCV knowledge scale was found to have good reliability and validity. The total Cronbach coefficient was 0.941(95% CI 0.936-0.945) and the Cronbach coefficients of disease nature and modes of transmission were 0.824 (95% CI 0.798-0.848), and 0.932 (95% CI 0.927-0.937), respectively.

The factor loading of each item of disease nature and modes of transmission was 0.779-0.985 and 0.648-0.967, respectively. The AVE values of the 2 factors were 0.514 and 0.640, respectively, and the CR values were all >0.7, demonstrating strong convergent validity of the questionnaire constructs (Table 2).

**Table 2:** Factor loading of each item and convergent validity and composite reliability measurement results.

Construct and item			Factor loading
**Disease nature (AVE^a^=0.514, CR^b^=0.769)**			
	HCV^c^ infection is usually asymptomatic and must be detected with testing.	0.822	
	Chronic HCV infection may develop into cirrhosis and liver cancer.	0.985	
	Hepatitis C can be cured.	0.779	
**Modes of transmission (AVE=0.640, CR=0.923)**			
	Can HCV be transmitted via kissing a person with HCV infection?	0.697	
	Can HCV be transmitted by sharing water and food with people with HCV infection?	0.648	
	Sexual contact with multiple partners will increase the risk of HCV transmission.	0.707	
	Can HCV be transmitted during pregnancy or birth in the case of a mother with HCV infection?	0.914	
	Can HCV be transmitted by using unsterilized syringes, needles, or surgical instruments?	0.967	
	Can HCV be transmitted by getting a tattoo/piercing?	0.768	

^a^AVE: average variance extracted.

^b^CR: composite reliability.

^c^HCV: hepatitis C virus.

The square roots of the AVE values were higher than the absolute values of the correlation coefficients between the disease nature and modes of transmission, indicating that the questionnaire had good discriminant validity (Table 3).

CFA results indicated that the 2-factor model with 9 items had a good fit: root mean square error of approximation (RMSEA)=0.034, comparative fit index (CFI)=0.953, Tucker-Lewis index (TLI)=0.954, and standardized root mean square residual (SRMR)=0.093.

**Table 3:** Correlation matrix of disease nature and modes of transmission.

Construct	Disease nature	Modes of transmission
Disease nature	0.717^a^	N/A^b^
Modes of transmission	0.675^c^	0.800^a^

^a^Square roots of the average variance extracted (AVE).

^b^N/A: not applicable.

^c^*P*<.05.

### Participant Knowledge of HCV

**Figure 2:**
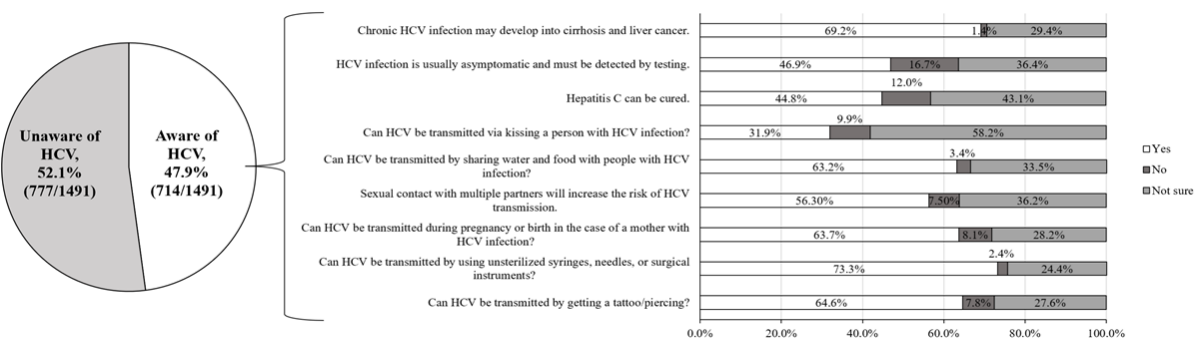
Awareness of HCV and knowledge regarding hepatitis C disease’s nature and modes of transmission. HCV: hepatitis C virus.

Less than half (714/1491, 47.9%) of the participants were aware of HCV. Of these, the proportions of participants who correctly answered the items related to disease nature ranged from 44.8% (320/714) to 69.2% (494/714) and of those who correctly answered the items related to modes of transmission ranged from 33.5% (239/714) to 73.3% (523/714). A detailed description of each item is presented in Figure 2.

Participants who were unaware of HCV were regarded as having poor knowledge. Overall, the proportion of participants who had poor, fair, and good knowledge was 63.4% (945/1491), 9.3% (139/1491), and 27.3% (407/1491), respectively.

### Participant HCV Testing Behavior and Willingness to Undergo HCV Screening

A total of 465 (31.2%) participants had ever undergone HCV testing. Of these, 4 (0.9%) were anti-HCV antibody positive and 288 (61.9%) were unaware of their results. Most participants underwent 1-time testing (419/465, 90.1%) and had undergone their last HCV test at a median of 16.0 (Q1, Q3: 10.0, 63.0) months previously.

**Figure 3:**
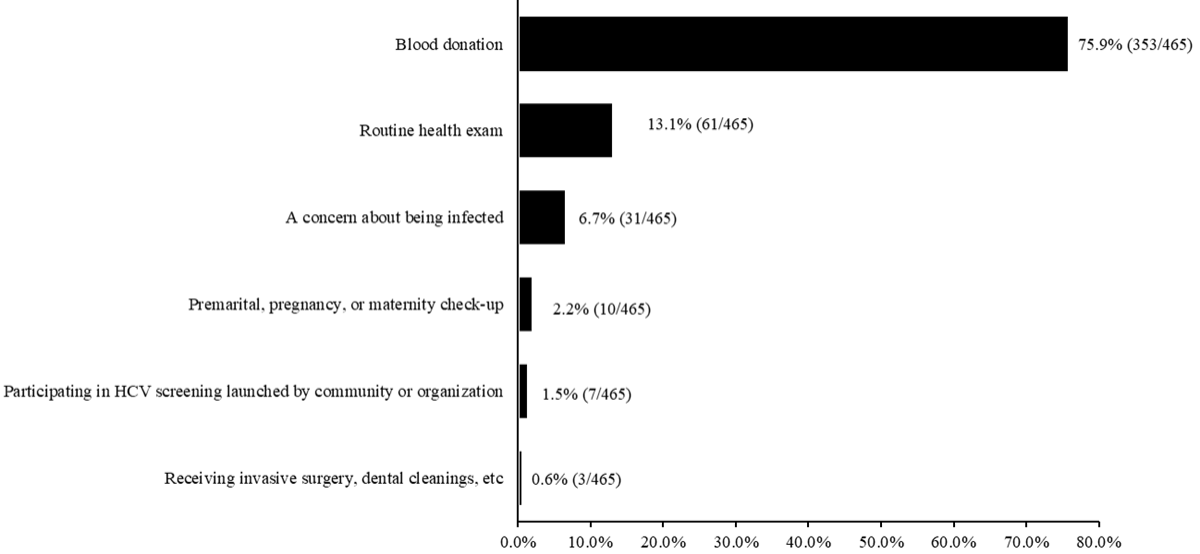
Participants' reasons for undergoing their last HCV test. HCV: hepatitis C virus.

The reasons for the last testing are shown in Figure 3. Most participants (353/465, 75.9%) were tested for HCV following blood donation, 13.1% (61/465) were tested during a routine health exam, and 6.7% (31/465) were tested due to a concern about being infected.

**Figure 4:**
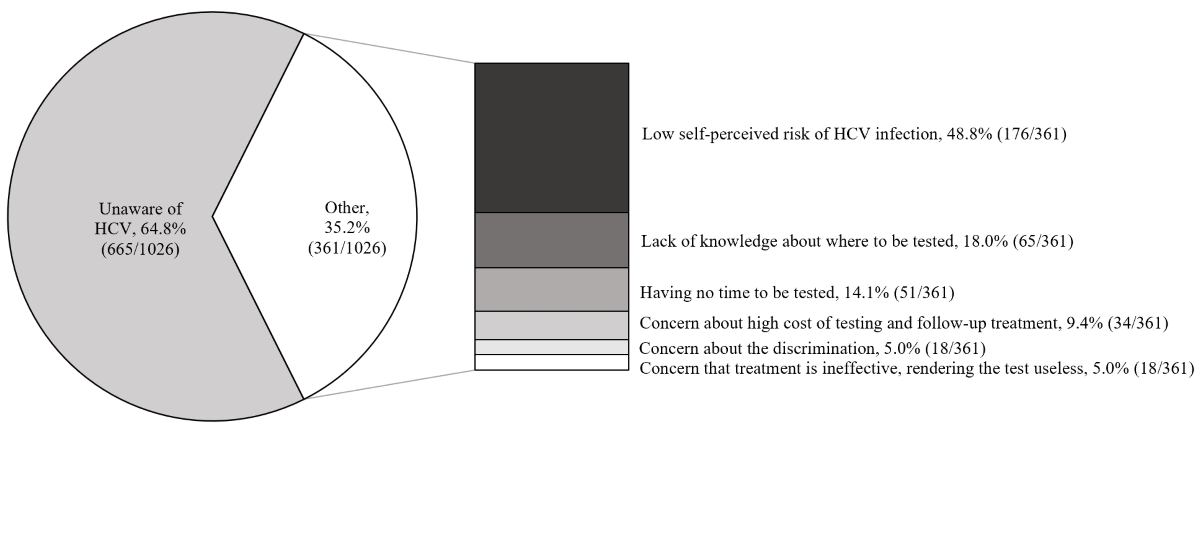
Participants' reasons for never undergoing an HCV test. HCV: hepatitis C virus.

The reasons the participants never underwent an HCV test are listed in Figure 4. The most common barrier against HCV testing was a lack of awareness about the virus (665/1026, 64.8%). Of the participants who were aware of HCV, the main reasons for not undergoing HCV testing included a low self-perceived risk of HCV infection (176/361, 48.8%), a lack of knowledge of where to be tested (65/361, 18.0%), and having no time to be tested (51/361, 14.1%).

**Figure 5:**
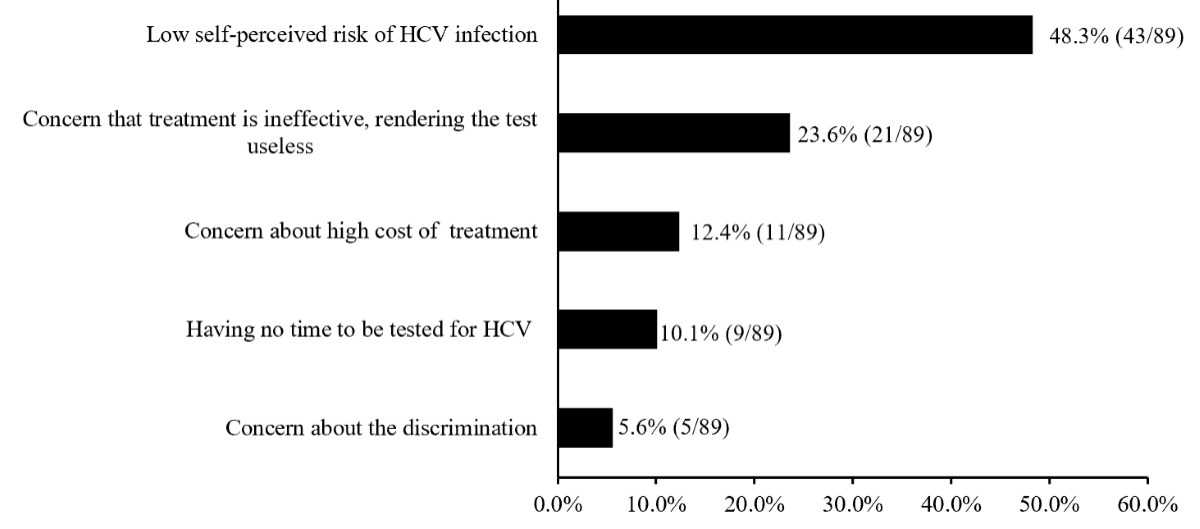
Participants' reasons for being unwilling to undergo HCV screening. HCV: hepatitis C virus.

Of the participants who never underwent HCV testing, 91.3% (937/1026) were willing to undergo HCV screening if universal screening was provided free of charge. The most common reasons participants were unwilling to undergo testing were a low self-perceived risk of HCV infection (43/89, 48.3%), followed by a concern that treatment is ineffective, rendering the test useless (21/89, 23.6%); see Figure 5.

### Factors Associated With HCV Knowledge

The correlation matrix did not show the existence of high collinearity between variables (Table S1 in Multimedia Appendix 2). Factors associated with HCV knowledge are shown in Tables 4 and 5. In the final multivariate ordinal logistic regression, participants aged ≥60 years (aOR=0.374, 95% CI 0.245-0.572), less educated (<7 years: aOR=0.607, 95% CI 0.401-0.919; 7-12 years: aOR=0.757, 95% CI 0.561-0.953), living in rural areas (aOR=0.625, 95% CI 0.472-0.830), located in West China (aOR=0.452, 95% CI 0.330-0.620), and currently drinking alcohol (aOR=0.680, 95% CI 0.520-0.890) had lower HCV knowledge scores. Participants with a blood donation history (aOR=1.758, 95% CI 1.376-2.245) and a family history of HBV or HCV infection (aOR=2.624, 95% CI 1.815-3.792) were more likely to have higher HCV knowledge scores.

**Table 4:** Factors associated with HCV^a^ knowledge (univariate analysis).

Characteristics		HCV knowledge level							Univariate analysis		
		Poor, n (%)		Fair, n (%)		Good, n (%)		cOR^b^ (95%CI)			*P* value^c^
**Age (years)**											
	<60	730 (59.3)	130 (10.6)		370 (30.1)		Reference			N/A^d^	
	≥60	215 (82.4)	9 (3.4)		37 (14.2)		0.322 (0.231-0.450)			<.001	
**Gender**											
	Male	430 (64.0)	62 (9.2)		180 (26.8)		Reference			N/A	
	Female	515 (62.9)	77 (9.4)		227 (27.7)		1.048 (0.851-1.290)			.655	
**Marital status**											
	Single/divorced/widowed	255 (59.2)	45 (10.4)		131 (30.4)		Reference			N/A	
	Married	690 (65.1)	94 (8.9)		276 (26.0)		0.786 (0.627-0.984)			.036	
**Years of education**											
	<7	257 (77.6)	18 (5.4)		56 (16.9)		0.339 (0.253-0.454)			<0.001	
	7-12	309 (70.2)	26 (5.9)		105 (23.9)		0.508 (0.397-0.650)			<0.001	
	>12	379 (52.6)	95 (13.2)		246 (34.2)		Reference			N/A	
**Ethnicity**											
	Han	894 (63.6)	126 (9.0)		385 (27.4)		Reference			N/A	
	Minority	51 (59.3)	13 (15.1)		22 (25.6)		1.101 (0.711-1.705)			.665	
**Occupation**											
	Students	76 (54.7)	19 (13.7)		44 (31.7)		Reference			N/A	
	Employed/self-employed	812 (63.1)	117 (9.1)		357 (27.8)		0.747 (0.530-1.053)			.097	
	Not working/unemployed	57 (86.4)	3 (4.5)		6 (9.1)		0.201 (0.092-0.435)			<.001	
**Residence**											
	Urban	472 (56.0)	99 (11.7)		272 (32.3)		Reference			N/A	
	Rural	473 (73.0)	40 (6.2)		135 (20.8)		0.492 (0.396-0.61)			<.001	
**Geographic region**											
	East	258 (54.2)	64 (13.4)		154 (32.4)		Reference			N/A	
	West	270 (74.2)	24 (6.6)		70 (19.2)		0.438 (0.327-0.585)			<.001	
	Central	417 (64.1)	51 (7.8)		183 (28.1)		0.714 (0.564-0.903)			.005	
**Alcohol** **consumption**											
	Never	569 (59.2)	110 (11.4)		282 (29.3)		Reference			N/A	
	Current	290 (73.6)	20 (5.1)		84 (21.3)		0.551 (0.427-0.710)			<.001	
	Ever	86 (63.2)	9 (6.6)		41 (30.1)		0.903 (0.628-1.296)			.579	
**History of blood donation**											
	Yes	181 (48.0)	54 (14.3)		142 (37.7)		2.189 (1.738-2.750)			<.001	
	No	764 (68.6)	85 (7.6)		265 (23.8)		Reference			N/A	
**Family history of HBV^e^ or HCV infection**											
	No	837 (64.1)	132 (10.1)		337 (25.8)		Reference			N/A	
	Yes	108 (58.4)	7 (3.8)		70 (37.8)		1.432 (1.058-1.938)			.020	

^a^HCV: hepatitis C virus.

^b^cOR: crude odds ratio.

^c^The proportional odds assumption was satisfied for each variable, with *P*>.05.

^d^N/A: not applicable.

^e^HBV: hepatitis B virus.

**Table 5:** Factors associated with HCV^a^ knowledge (multivariate analysis).

Characteristics		HCV knowledge level							Multivariate analysis		
		Poor, n (%)		Fair, n (%)		Good, n (%)		aOR^b^ (95%CI)			*P* value^c^
**Age (years)**											
	<60	730 (59.3)	130 (10.6)		370 (30.1)		Reference			N/A^d^	
	≥60	215 (82.4)	9 (3.4)		37 (14.2)		0.374 (0.245-0.572)			<.001	
**Gender**											
	Male	430 (64.0)	62 (9.2)		180 (26.8)		N/A			N/A	
	Female	515 (62.9)	77 (9.4)		227 (27.7)		N/A			N/A	
**Marital status**											
	Single/divorced/widowed	255 (59.2)	45 (10.4)		131 (30.4)		Reference			N/A	
	Married	690 (65.1)	94 (8.9)		276 (26.0)		0.999 (0.759-1.316)			.978	
**Years of education**											
	<7	257 (77.6)	18 (5.4)		56 (16.9)		0.607 (0.401-0.919)			.007	
	7-12	309 (70.2)	26 (5.9)		105 (23.9)		0.757 (0.561-0.953)			.022	
	>12	379 (52.6)	95 (13.2)		246 (34.2)		Reference			N/A	
**Ethnicity**											
	Han	894 (63.6)	126 (9.0)		385 (27.4)		N/A			N/A	
	Minority	51 (59.3)	13 (15.1)		22 (25.6)		N/A			N/A	
**Occupation**											
	Students	76 (54.7)	19 (13.7)		44 (31.7)		Reference			N/A	
	Employed/self-employed	812 (63.1)	117 (9.1)		357 (27.8)		0.828 (0.550-1.246)			.351	
	Not working/unemployed	57 (86.4)	3 (4.5)		6 (9.1)		0.612 (0.096-1.128)			.111	
**Residence**											
	Urban	472 (56.0)	99 (11.7)		272 (32.3)		Reference			N/A	
	Rural	473 (73.0)	40 (6.2)		135 (20.8)		0.625 (0.472-0.830)			.004	
**Geographic region**											
	East	258 (54.2)	64 (13.4)		154 (32.4)		Reference			N/A	
	West	270 (74.2)	24 (6.6)		70 (19.2)		0.452 (0.330-0.620)			<.001	
	Central	417 (64.1)	51 (7.8)		183 (28.1)		0.974 (0.744-1.275)			.848	
**Alcohol** **consumption**											
	Never	569 (59.2)	110 (11.4)		282 (29.3)		Reference			N/A	
	Current	290 (73.6)	20 (5.1)		84 (21.3)		0.680 (0.520-0.890)			.015	
	Ever	86 (63.2)	9 (6.6)		41 (30.1)		0.844 (0.574-1.243)			.485	
**History of blood donation**											
	Yes	181 (48.0)	54 (14.3)		142 (37.7)		1.758 (1.376-2.245)			<.001	
	No	764 (68.6)	85 (7.6)		265 (23.8)		Reference			N/A	
**Family history of HBV^e^or HCV infection**											
	No	837 (64.1)	132 (10.1)		337 (25.8)		Reference			N/A	
	Yes	108 (58.4)	7 (3.8)		70 (37.8)		2.624 (1.815-3.792)			<.001	

^a^HCV: hepatitis C virus.

^b^aOR: adjusted odds ratio.

^c^The proportional odds assumption was satisfied for each variable, with *P*>.05.

^d^N/A: not applicable.

^e^HBV: hepatitis B virus.

### Factors Associated With the Uptake of HCV Testing

The correlation matrix did not show the existence of high collinearity between variables (Table S1 in Multimedia Appendix 2). Factors associated with the uptake of HCV testing are presented as Tables 6 and 7. In the final multivariate logistic regression, participants who were female (aOR=0.623, 95% CI 0.478-0.813), were less educated (<7 years: aOR=0.210, 95% CI 0.139-0.316; 7-12 years: aOR=0.693, 95% CI 0.508-0.946), lived in rural areas (aOR=0.690, 95% CI 0.548-0.868), were located in West China (aOR=0.434, 95% CI 0.306-0.617), currently drank alcohol (aOR=0.441, 95% CI 0.318-0.613), and had poor HCV knowledge (aOR=0.353, 95% CI 0.270-0.461) had a lower odds of being previously tested for HCV. Participants with a family history of HBV or HCV infection (aOR=5.126, 95% CI 3.457-7.600) had higher odds of having undergone HCV testing. After limiting participants without a history of blood donation, factors associated with the uptake of HCV testing remained similar, except that a few variables lost their statistical significance due to the reduced sample size (Table S2 in Multimedia Appendix 3).

**Table 6:** Factors associated with the uptake of HCV^a^ testing (univariate analysis).

Characteristics		Uptake of HCV testing				Univariate analysis		
		No, n (%)		Yes, n (%)		cOR^b^ (95%CI)		*P* value
**Age (years)**								
	<60	838(68.1)	392(31.9)		Reference		N/A^c^	
	≥60	188(72.0)	73(28.0)		0.830(0.617–1.115)		.217	
**Gender**								
	Male	447 (66.5)	225 (33.5)		Reference		N/A	
	Female	579 (70.7)	240 (29.3)		0.823 (0.661-1.025)		.083	
**Marital status**								
	Single/divorced/widowed	285 (66.1)	146 (33.9)		Reference		N/A	
	Married	741 (69.9)	319 (30.1)		0.840 (0.661-1.067)		.153	
**Years of education**								
	<7	274 (82.8)	57 (17.2)		0.321 (0.232-0.443)		<.001	
	7-12	315 (71.6)	125 (28.4)		0.613 (0.474-0.791)		<.001	
	>12	437 (60.7)	283 (39.3)		Reference		N/A	
**Ethnicity**								
	Han	969 (69.0)	436 (31.0)		Reference		N/A	
	Minority	57 (66.3)	29 (33.7)		1.131 (0.71-1.793)		.601	
**Occupation**								
	Students	86 (61.9)	53 (38.1)		Reference		N/A	
	Employed/self-employed	888 (69.1)	398 (30.9)		0.727 (0.506-1.044)		.085	
	Not working/unemployed	52 (78.8)	14 (21.2)		0.437 (0.221-0.864)		.017	
**Residence**								
	Urban	554 (65.7)	289 (34.3)		Reference		N/A	
	Rural	472 (72.8)	176 (27.2)		0.715 (0.571-0.894)		.003	
**Geographic region**								
	East	301 (63.2)	175 (36.8)		Reference		N/A	
	West	284 (78.0)	80 (22.0)		0.484 (0.355-0.660)		<.001	
	Central	441 (67.7)	210 (32.3)		0.819 (0.638-1.05)		.115	
**Alcohol** **consumption**								
	Never	626 (65.1)	335 (34.9)		Reference		N/A	
	Current	320 (81.2)	74 (18.8)		0.432 (0.324-0.574)		<.001	
	Ever	80 (58.8)	56 (41.2)		1.308 (0.906-1.886)		.151	
**Family history of HBV^d^ or HCV infection**								
	Yes	937 (71.7)	369 (28.3)		Reference		N/A	
	No	89 (48.1)	96 (51.9)		2.738 (2.003-3.7)		<.001	
**HCV knowledge level**								
	Poor	752 (79.6)	193 (20.4)		0.248 (0.193-0.318)		<.001	
	Fair	74 (53.2)	65 (46.8)		0.849 (0.577-1.248)		.404	
	Good	200 (49.1)	207 (50.9)		Reference		N/A	

^a^HCV: hepatitis C virus.

^b^cOR: crude odds ratio.

^c^N/A: not applicable.

^d^HBV: hepatitis B virus.

**Table 7:** Factors associated with the uptake of HCV^a^ testing (multivariate analysis).

Characteristics		Uptake of HCV testing				Multivariate analysis		
		No, n (%)		Yes, n (%)		aOR^b^ (95%CI)		*P* value
**Age (years)**								
	<60	838(68.1)	392(31.9)		N/A^c^		N/A	
	≥60	188(72.0)	73(28.0)		N/A		N/A	
**Gender**								
	Male	447 (66.5)	225 (33.5)		Reference		N/A	
	Female	579 (70.7)	240 (29.3)		0.623 (0.478-0.813)		<.001	
**Marital status**								
	Single/divorced/widowed	285 (66.1)	146 (33.9)		N/A		N/A	
	Married	741 (69.9)	319 (30.1)		0.811 (0.635-1.037)		.152	
**Years of education**								
	<7	274 (82.8)	57 (17.2)		0.210 (0.139-0.316)		<.001	
	7-12	315 (71.6)	125 (28.4)		0.693 (0.508-0.946)		.021	
	>12	437 (60.7)	283 (39.3)		Reference		N/A	
**Ethnicity**								
	Han	969 (69.0)	436 (31.0)		N/A		N/A	
	Minority	57 (66.3)	29 (33.7)		N/A		N/A	
**Occupation**								
	Students	86 (61.9)	53 (38.1)		Reference		N/A	
	Employed/self-employed	888 (69.1)	398 (30.9)		0.831 (0.364-1.896)		.205	
	Not working/unemployed	52 (78.8)	14 (21.2)		0.726 (0.457-1.154)		.517	
**Residence**								
	Urban	554 (65.7)	289 (34.3)		Reference		N/A	
	Rural	472 (72.8)	176 (27.2)		0.690 (0.548-0.868)		<.001	
**Geographic region**								
	East	301 (63.2)	175 (36.8)		Reference		N/A	
	West	284 (78.0)	80 (22.0)		0.434 (0.306-0.617)		<.001	
	Central	441 (67.7)	210 (32.3)		0.999 (0.753-1.325)		.996	
**Alcohol consumption**								
	Never	626 (65.1)	335 (34.9)		Reference		N/A	
	Current	320 (81.2)	74 (18.8)		0.441 (0.318-0.613)		<.001	
	Ever	80 (58.8)	56 (41.2)		1.313 (0.868-1.984)		.197	
**Family history of HBV^d^ or HCV infection**								
	Yes	937 (71.7)	369 (28.3)		Reference		N/A	
	No	89 (48.1)	96 (51.9)		5.126 (3.457-7.600)		<.001	
**HCV knowledge level**								
	Poor	752 (79.6)	193 (20.4)		0.353 (0.270-0.461)		<.001	
	Fair	74 (53.2)	65 (46.8)		0.920 (0.611-1.385)		.689	
	Good	200 (49.1)	207 (50.9)		Reference		N/A	

^a^HCV: hepatitis C virus.

^b^aOR: adjusted odds ratio.

^c^N/A: not applicable.

^d^HBV: hepatitis B virus.

## Discussion

### Principal Findings

HCV is a serious public health problem in both China and worldwide. To the best of our knowledge, this is the first study to assess the HCV knowledge and testing behavior of the general Chinese population. The findings inform the development and improvement of HCV-related health education and screening strategies.

HCV knowledge, even at a basic level, was low in the general Chinese population included in this survey. Less than half of the participants had ever heard of HCV, which is lower than that reported in high-income countries. In South Korea, for example, 56.4% of the general population was aware of HCV [22]. The lack of health education in the general population of China is an important reason for the low rate of disease awareness [23]. Although China issued “Guidelines for the Prevention and Treatment of Hepatitis C” in 2004, this document only emphasizes the need for HCV health education in high-risk groups [13,24]. After evaluating HCV knowledge among participants in this study, several concerns were found to be worth noting. First, participants had particularly low awareness of treatment outcomes, a finding that could delay or prevent treatment initiation. Only 44.8% of survey respondents who were aware of HCV believed that the disease could be cured. This may be because prior to the approval of DAAs, the traditional treatment for HCV was a combined injection of peg-interferon-α and ribavirin, and the sustained virologic response (SVR) to this medication was only about 52% (95% CI 34%-69%) [25-27]. Although the more recent approval of DAAs has meant that >95% of patients can now achieve an SVR [11] , the success of this treatment is not widely known. Second, participants had low knowledge of sexual or household transmission of HCV. For example, of the participants who had ever heard of HCV, >60% were aware that HCV can be transmitted via blood or from mother to child. In contrast, 63.2% believed incorrectly that people could become infected by sharing water or food with a person with HCV infection. This misunderstanding could result in discrimination against individuals with HCV infection, thereby preventing them from undergoing testing and engaging in normal social and work activities. In addition, slightly over half of the participants who were aware of HCV recognized that sexual contact with multiple partners can increase the risk of transmission. Although sexual transmission in monogamous heterosexual relationships is rare, we still included it as a question on HCV knowledge. This is mainly because unsafe sexual contact is an important route of HCV transmission among MSM or people with HIV infection [28,29]. In this study, MSM or HIV infection was not included as a variable, so it was not possible to assess knowledge of the sexual transmission of HCV in this population. In addition, unsafe sexual contact increases the risk of other infectious diseases, including HIV, HBV, and syphilis. Thus, we hope that the negative public attitude toward unsafe sexual contact is beneficial for the prevention of multiple infections, including HCV. These findings indicate that public health education is urgently needed in China, especially regarding HCV treatment outcomes and modes of transmission.

About one-third of the general population (31.2%) included in this study reported ever undergoing HCV testing, which was lower than that reported in high-income countries. In Italy, for example, 43.4% of the general population declared that it has undergone HCV testing in the past [30]. Moreover, this study found that 75.9% of the survey respondents who had undergone HCV testing did so during blood donation. This might be related to the increased focus on the safety of blood products. Since 1993, the Chinese government has had a policy to screen blood donors and has enacted related laws to screen all donors for anti-HCV antibodies; in 2015, China began screening blood donors who were anti-HCV negative for HCV RNA [20]. However, of participants without a history of blood donation, only 7.9% had ever been tested for HCV, most often during a routine health exam. In addition, most of those tested for HCV were unaware of their results, which could lead to delayed diagnosis and treatment. In fact, most anti-HCV antibody–positive patients often get no opportunity to undergo early treatment due to limited options for HCV consultation and referral in China. According to a survey conducted among patients with HCV in 76 hospitals in China, only 34.9% underwent further HCV RNA testing and 12.2% underwent antiviral treatment [31]. Furthermore, a lack of knowledge of where to be tested and having no time to be tested were the important reasons participants who were aware of HCV did not undergo HCV testing. Self-testing for HCV antibodies, where people collect their own specimen, perform a simple rapid test, and interpret the result, has been recommended by WHO as an additional testing approach to increase HCV testing coverage since 2021 [32]. Self-testing offers privacy and convenience. A systematic review and meta-analysis also suggested that HCV self-testing is feasible and well accepted [33]. In China, evaluation of the cost-effectiveness of HCV self-testing is needed to implement and scale-up this program nationwide. Nevertheless, it is critically important to expand HCV knowledge in the general population and strengthen access to testing and standardized treatment in China.

Despite the overall prevalence of HCV antibody–positive individuals in the general Chinese population being reported as only 0.91%, prevalence was shown to increase with advancing age, peaking at 3.95% among people >65 years of age [10]. Thus, older people may be the key population for HCV testing in China. However, this study found that the HCV testing rate is not significantly higher among the elderly, suggesting that targeted interventions are required for the older population. Low awareness of HCV and a low self-perceived risk of HCV infection were the most reported barriers against HCV testing, and the elderly were also shown to have low HCV knowledge. In addition to strengthening publicity and education, birth cohort screening is recommended. WHO suggests that universal screening be implemented in populations with an HCV antibody seroprevalence of >2%-5% and indicates that birth cohort screening of older adults who have high seroprevalence (eg, people born between 1945 and 1965 in the United States) in settings with a low general prevalence is cost-effective [34]. This study found that people more than 60 years old had a high willingness (162/188, 86.2%) to undergo HCV testing if universal screening was provided free of charge. Thus, it may be feasible to perform universal birth cohort screening. However, cost-effectiveness analysis is needed to determine the optimal birth cohort for HCV screening in order to achieve the goal of eliminating HCV in China.

This study assumed that all participants with a history of blood donation were tested for HCV. Under this assumption, we found that those who were less educated, living in rural areas, and located in West China had less HCV knowledge and testing uptake. West China is a less developed region compared to East and Central China [35]. These findings are in line with previous studies showing that individuals with poor education and living in less developed areas have less access to health care innovations [36]. In addition to sociodemographic characteristics, the findings of this study revealed that people who currently drink alcohol have low HCV knowledge and a low likelihood of undergoing testing and those with a history of blood donation and a family history of viral hepatitis have higher knowledge and testing. Although alcohol use is not a risk factor for HCV, it is believed to have a deleterious impact on the course of hepatitis C disease [37], for example, to promote HCV infection persistence, cytotoxicity, and oxidative stress. Thus, compared to nondrinkers, alcohol drinkers may be in greater need for HCV education and testing interventions to prevent and promote the early treatment of HCV. Blood donors tend to be well educated and may receive HCV education in blood donation centers [38]. People with a family history of HBV or HCV may be prompted to seek health care due to a concern about exposure. This study also showed that participants with low HCV knowledge were less likely to initiate HCV testing, suggesting that improving HCV knowledge may increase testing rates. In summary, HCV-related health education should be prioritized for those who are less educated, located in less-developed areas, drink alcohol, and may otherwise have limited health awareness.

It is worth noting that the HCV knowledge of women was not significantly lower than that of men but their testing rate was significantly lower. Further analysis revealed that in this survey, compared to men, women had a higher proportion of low self-perceived risk of HCV infection (men: 50/447, 11.8%; women: 126/579, 22.8%) and a lack of time to be tested (men: 11/447, 2.5%; women: 40/579, 6.9%). However, women are at higher risk of both transmitting and acquiring HCV through sexual and vertical (mother to child during childbirth) transmission [39,40]. Thus, suitable and effective strategies should be implemented for women, including help in accessing health care services.

### Limitations

This study has several limitations. First, since WeChat was used to disseminate the questionnaire, only those with access to this social media platform were included in the study. This may have resulted in an insufficient representation of the general population and limited the conclusions. Generally, people without access to this social media platform are more likely to be less educated and located in low-income areas [41]; thus, it can be inferred that HCV knowledge and the testing rate of the general Chinese population would be lower than that of this survey. Second, the data presented in this study were self-reported and thus might be subject to recall and social bias. Third, this was a cross-sectional study; thus, no causal relationship could be concluded from the findings. Finally, only some indicators of HCV knowledge and the uptake of HCV testing were investigated.

### Conclusion

This study highlights that the general population of China has limited HCV knowledge and a low HCV testing rate. There is an urgent need to design and distribute education information to enhance HCV knowledge and provide targeted information regarding treatment outcomes and all possible modes of transmission. Increased referral to standardized treatment and scaled-up HCV screening, including birth cohort screening for older adults at higher risk of HCV transmission and infection, is also recommended. In addition, health education and interventions should be prioritized for women, those with less education, those living in less-developed areas, and those who currently drink alcohol.

## Appendix

Multimedia Appendix 1 The geographical distribution of the included participants.

Multimedia Appendix 2 Correlation matrix of the included variables.

Multimedia Appendix 3 Factors associated with the uptake of HCV testing in participants without a history of blood donation.

## Abbreviations

aOR: adjusted odds ratio
AVE: average variance extracted
CFA: confirmatory factor analysis
cOR: crude odds ratio
CR: composite reliability
DAA: direct acting antiviral
HBV: hepatitis B virus
HCV: hepatitis C virus
MSM: men who have sex with men
SVR: sustained virologic response
WHO: World Health Organization
