# Supplementary figures and images for: Hepatitis C Knowledge and Self-Reported Testing Behavior in the General Population in China: Online Cross-Sectional Survey
Source: JMIR Public Health Surveill. 2023 Dec 11;9:e39472. doi: 10.2196/39472 (PMC10760629; doi:10.2196/39472)

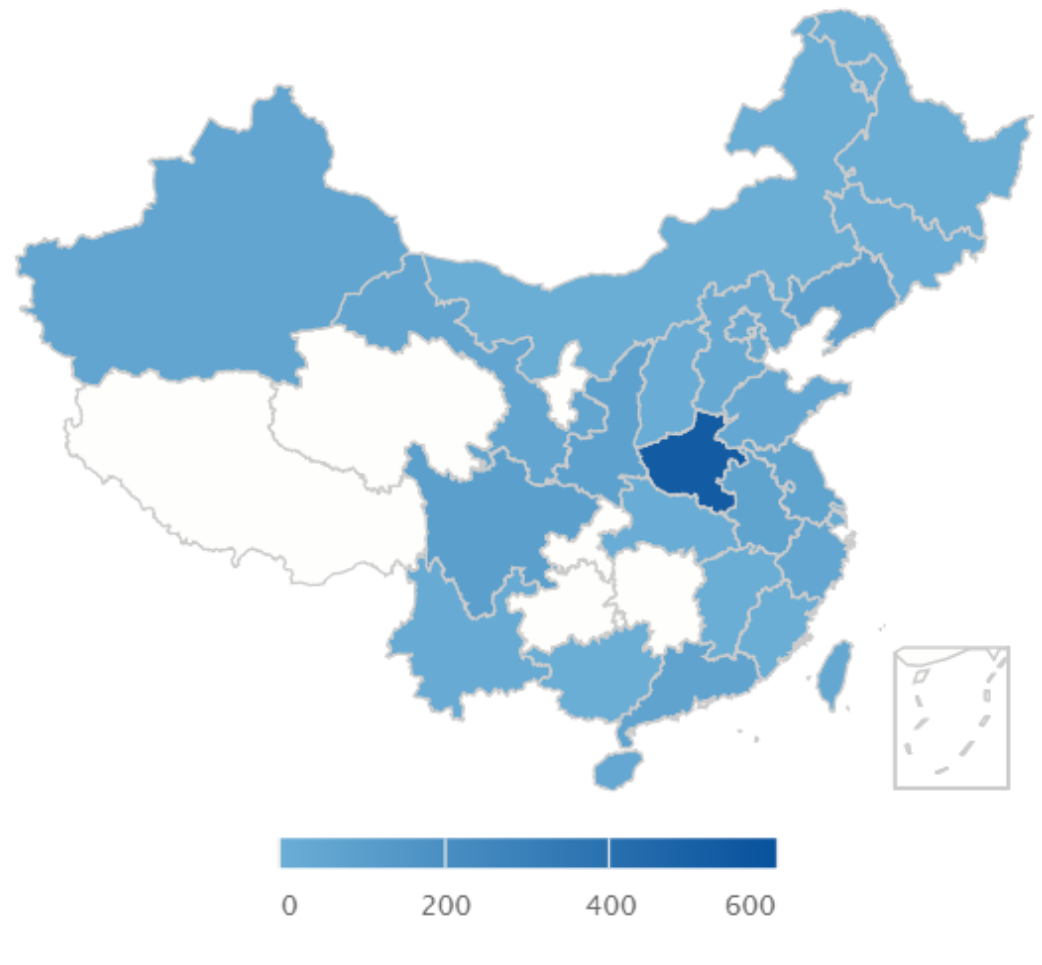

Supplement: Multimedia Appendix 1 [file publichealth_v9i1e39472_app1.png]
