# Supplementary material for: Hepatitis C Knowledge and Self-Reported Testing Behavior in the General Population in China: Online Cross-Sectional Survey
Source: JMIR Public Health Surveill. 2023 Dec 11;9:e39472. doi: 10.2196/39472 (PMC10760629; doi:10.2196/39472)
Supplement: Multimedia Appendix 2 [file publichealth_v9i1e39472_app2.pdf]

Table S1 Correlation matrix of the included variables

	**Age (years old)**	**Gender**	**Marital status**	**Years of education**	**Ethnic**	**Occupation**	**Residence**	**Geographic region**	**Alcohol drinking**	**History of blood donation**	**Family history of HBV or HCV infection**	**HCV knowledge level**
**Age (years old)**	1.000	-0.069	0.095	-0.520	-0.114	0.095	0.383	-0.185	-0.034	0.053	0.362	0.046
**Gender**	-0.069	1.000	-0.030	-0.023	0.184	-0.007	0.071	0.011	-0.382	0.062	0.079	0.071
**Marital status**	0.095	-0.030	1.000	-0.368	-0.191	0.390	0.156	-0.164	-0.015	0.041	-0.029	0.037
**Years of education**	-0.520	-0.023	-0.368	1.000	0.208	-0.304	-0.543	0.243	-0.004	-0.164	-0.192	-0.159
**Ethnic**	-0.114	0.184	-0.191	0.208	1.000	-0.077	-0.078	0.094	-0.125	0.005	0.081	-0.045
**Occupation**	0.095	-0.007	0.390	-0.304	-0.077	1.000	0.113	-0.025	0.010	0.065	0.011	0.070
**Residence**	0.383	0.071	0.156	-0.543	-0.078	0.113	1.000	-0.314	-0.063	0.074	0.204	0.131
**Geographic region**	-0.185	0.011	-0.164	0.243	0.094	-0.025	-0.314	1.000	0.003	-0.056	-0.046	-0.009
**Alcohol drinking**	-0.034	-0.382	-0.015	-0.004	-0.125	0.010	-0.063	0.003	1.000	-0.006	-0.101	0.135
**History of blood donation**	0.053	0.062	0.041	-0.164	0.005	0.065	0.074	-0.056	-0.006	1.000	-0.043	0.261
**Family history of HBV or HCV infection**	0.362	0.079	-0.029	-0.192	0.081	0.011	0.204	-0.046	-0.101	-0.043	1.000	-0.091
**HCV knowledge level**	0.046	0.071	0.037	-0.159	-0.045	0.070	0.131	-0.009	0.135	0.261	-0.091	1.000
