# Supplementary material for: Hepatitis C Knowledge and Self-Reported Testing Behavior in the General Population in China: Online Cross-Sectional Survey
Source: JMIR Public Health Surveill. 2023 Dec 11;9:e39472. doi: 10.2196/39472 (PMC10760629; doi:10.2196/39472)
Supplement: Multimedia Appendix 3 [file publichealth_v9i1e39472_app3.pdf]

Table S2 Factors associated with the uptake of HCV testing in participants without a history of blood donation

Characteristics	Uptake of HCV testing		Univariate analysis		Multivariate analysis	
	No n(%)	Yes n(%)	cOR(95%CI)	*P*	aOR(95%CI)	*P*
**Age (years old)**						
<60	838(92.5)	68(7.5)	Reference			
≥60	188(90.4)	20(9.6)	1.311(0.777-2.212)	0.310		
**Gender**						
Male	447(92.7)	35(7.3)	Reference			
Female	579(91.6)	53(8.4)	1.169(0.749-1.823)	0.491		
**Marital status**						
Single/divorced/widowed	285(91.9)	25(8.1)	Reference			
married	741(92.2)	63(7.8)	0.969(0.597-1.570)	0.898		
**Years of education**						
<7	274(98.9)	3(1.1)	0.104(0.032-0.337)	<0.001	0.020(0.004-0.104)	<0.001
7-12	315(89.0)	39(11.0)	1.176(0.749-1.845)	0.480	1.285(0.236-3.225)	0.084
>12	437(90.5)	46(9.5)	Reference		Reference	
**Ethnic**						
Han	969(92.4)	80(7.6)	Reference			
Minority	57(87.7)	8(12.3)	1.700(0.783-3.687)	0.179		
**Occupation**						
Students	86(91.5)	8(8.5)	Reference			
Employed/Self-employed	888(92.0)	77(8.0)	0.932(0.435-1.995)	0.856		
Not working/Unemployed	52(94.5)	3(5.5)	0.620(0.157-2.442)	0.495		
**Residence**						
Urban	554(91.4)	52(8.6)	Reference			
Rural	472(92.9)	36(7.1)	0.813(0.522-1.264)	0.358		
**Geographic region**						
East	301(91.5)	28(8.5)	Reference		Reference	
West	284(96.6)	10(3.4)	0.379(0.180-0.793)	0.010	0.705(0.259-1.920)	0.494
Central	441(89.8)	50(10.2)	1.219(0.750-1.980)	0.424	1.855(0.672-4.233)	0.162
**Alcohol drinking**						
Never	626(89.2)	76(10.8)	Reference		Reference	
Current	320(97.3)	9(2.7)	0.232(0.114-0.460)	<0.001	0.358(0.147-0.871)	<0.001
Ever	80(96.4)	3(3.6)	0.309(0.095-1.002)	0.051	0.526(0.465-1.001)	0.057
**Family history of HBV or HCV infection**						
No	937(95.1)	48(4.9)	Reference		Reference	
Yes	89(69.0)	40(31.0)	8.773(5.469-14.07)	<0.001	10.896(6.597-15.195)	<0.001
**HCV knowledge level**						
Poor	752(98.4)	12(1.6)	0.049(0.026-0.092)	<0.001	0.017(0.006-0.052)	<0.001
Fair	74(87.1)	11(12.9)	0.457(0.220-0.914)	0.027	0581(0.251-1.347)	0.206

Characteristics	Uptake of HCV testing		Univariate analysis		Multivariate analysis	
	**No n(%)**	**Yes n(%)**	**cOR(95%CI)**	***P***	**aOR(95%CI)**	***P***
Good	200(75.5)	65(24.5)	Reference		Reference	

cOR: crude odds ratio; aOR: adjusted odds ratio; CI: confidence interval.
HBV: Hepatitis B virus; HCV: Hepatitis C virus.
